# Altered Expression of Mitoferrin and Frataxin, Larger Labile Iron Pool and Greater Mitochondrial DNA Damage in the Skeletal Muscle of Older Adults

**DOI:** 10.3390/cells9122579

**Published:** 2020-12-02

**Authors:** Anna Picca, Sunil K. Saini, Robert T. Mankowski, George Kamenov, Stephen D. Anton, Todd M. Manini, Thomas W. Buford, Stephanie E. Wohlgemuth, Rui Xiao, Riccardo Calvani, Hélio José Coelho-Júnior, Francesco Landi, Roberto Bernabei, David A. Hood, Emanuele Marzetti, Christiaan Leeuwenburgh

**Affiliations:** 1 Fondazione Policlinico Universitario “Agostino Gemelli” IRCCS, 00168 Rome, Italy; anna.picca@guest.policlinicogemelli.it (A.P.); riccardo.calvani@guest.policlinicogemelli.it (R.C.); francesco.landi@unicatt.it (F.L.); roberto.bernabei@unicatt.it (R.B.); 2 Aging Research Center, Department of Neurobiology, Care Sciences and Society, Karolinska Institutet and Stockholm University, SE-171 77 Stockholm, Sweden; 3 Department of Aging and Geriatric Research, Institute on Aging, University of Florida, Gainesville, FL 32611, USA; sunil.saini@ufl.edu (S.K.S.); r.mankowski@ufl.edu (R.T.M.); santon@ufl.edu (S.D.A.); tmanini@ufl.edu (T.M.M.); steffiw@ufl.edu (S.E.W.); rxiao@ufl.edu (R.X.); cleeuwen@ufl.edu (C.L.); 4 Department of Geological Sciences, University of Florida, Gainesville, FL 32605, USA; kamenov@ufl.edu; 5 Department of Medicine, University of Alabama at Birmingham, Birmingham, AL 35205, USA; twbuford@uabmc.edu; 6 Institute of Internal Medicine and Geriatrics, Università Cattolica del Sacro Cuore, 00168 Rome, Italy; coelhojunior@hotmail.com.br; 7 Muscle Health Research Centre, School of Kinesiology and Health Science, York University, Toronto, ON M3J 1P3, Canada; dhood@yorku.ca

**Keywords:** autophagy, iron dyshomeostasis, iron metabolism, iron isotopes, mitochondria, mitochondrial quality control, mitophagy, muscle aging, oxidative stress, physical performance

## Abstract

Mitochondrial dysfunction and iron (Fe) dyshomeostasis are invoked among the mechanisms contributing to muscle aging, possibly via a detrimental mitochondrial–iron feed-forward loop. We quantified the labile Fe pool, Fe isotopes, and the expression of mitochondrial Fe handling proteins in muscle biopsies obtained from young and older adults. The expression of key proteins of mitochondrial quality control (MQC) and the abundance of the mitochondrial DNA common deletion (mtDNA^4977^) were also assessed. An inverse association was found between total Fe and the heavier Fe isotope (^56^Fe), indicating an increase in labile Fe abundance in cells with greater Fe content. The highest levels of labile Fe were detected in old participants with a Short Physical Performance Battery (SPPB) score ≤ 7 (low-functioning, LF). Protein levels of mitoferrin and frataxin were, respectively, higher and lower in the LF group relative to young participants and older adults with SPPB scores ≥ 11 (high-functioning, HF). The mtDNA^4977^ relative abundance was greater in old than in young participants, regardless of SPPB category. Higher protein levels of Pink1 were detected in LF participants compared with young and HF groups. Finally, the ratio between lipidated and non-lipidated microtubule-associated protein 1A/1B-light chain 3 (i.e., LC3B II/I), as well as p62 protein expression was lower in old participants regardless of SPPB scores. Our findings indicate that cellular and mitochondrial Fe homeostasis is perturbed in the aged muscle (especially in LF older adults), as reflected by altered levels of mitoferrin and frataxin, which, together with MQC derangements, might contribute to loss of mtDNA stability.

## 1. Introduction

The preservation of mobility and functional independence in older adults has become a clinical and public health priority, as well as a major goal of the National Institute on Aging and the National Institutes of Health strategic plan for research on aging [1]. Our work and that of others have shown that low-functioning (LF) older adults have a more rapid functional decline compared with those who are high-functioning (HF) [2,3,4,5,6,7]. Habitual walking speed and performance on standard measures of physical function can be considered a composite measure of healthy aging and predict mobility disability and all-cause mortality [3,4,5,6,7]. However, the biological mechanisms underlying the accelerated functional loss in LF older adults remain poorly understood, and few strategies are currently available to prevent this decline.

Mitochondrial dysfunction in skeletal muscle is invoked as a major factor underlying the onset and progression of functional decline during aging [8,9,10,11]. Yet, the molecular determinants underlying mitochondrial dysfunction in muscle and the associated functional decline are presently unclear. Notwithstanding, alterations in mitochondrial iron (Fe) handling in skeletal myocytes have been proposed as a mechanism leading to organelle dysfunction and muscle atrophy [12]. Indeed, variants in genes involved in the regulation of Fe handling were found to be associated with healthspan and physical performance in older adults [13,14].

The majority of body Fe is sequestered as heme Fe into functional biomolecules, including hemoglobin, myoglobin, cytochromes, and heme thiolates [15]. A smaller fraction of non-heme Fe exists as metallic ion serving as an enzyme cofactor or bound to cytosolic ferritin and hemosiderin to form Fe reserves [15]. Non-heme Fe is also an integral part of transferrin and is core to mitochondrial electron transport chain (ETC) complexes within Fe-sulfur clusters (ISCs) [16,17]. Of all non-heme Fe, approximately 5% exists as a chelatable Fe fraction and feeds a labile Fe pool. The biological relevance of this fraction resides in its chemistry involving ferrous (Fe^2+^) and ferric (Fe^3+^) ions. Fe^2+^ participates in Fenton reactions and produce highly reactive radicals, holding the potential of generating protein, lipid and nucleic acid oxidative adducts which are detrimental to the cell [18,19]. The reduction of Fe^3+^ to Fe^2+^ in the human body is accompanied by Fe isotope fractionation resulting in enrichment of the lighter isotope (^54^Fe) in the reduced Fe fraction [20,21]. Therefore, the isotope composition of the cellular Fe pool may represent a “surrogate” indicator of the cell’s redox status. 

The redistribution of non-heme Fe across tissues is regulated by the defensin-like hormone hepcidin via binding and subsequent degradation of the Fe export protein ferroportin [22,23]. Cellular Fe import, instead, occurs via a transferrin receptor (TfR) and is highly responsive to intracellular Fe levels. The two mitochondrial proteins, mitoferrin and frataxin, also participate in intracellular Fe handling. Mitoferrin, which is located at the inner mitochondrial membrane, regulates mitochondrial Fe import, while frataxin is pivotal for mitochondrial Fe storage. Studies in preclinical models have established that frataxin is also involved in heme biosynthesis, ISC cluster assembly, and aconitase repair [24,25]. Frataxin deficiency in humans results in mitochondrial Fe overload and Friedreich’s ataxia, a major inheritable neurodegenerative disorder [26].

The skeletal muscle is the major bodily reservoir of non-heme Fe. The latter accumulates in muscles with aging, which has been linked to oxidative damage to biomolecules and organelles, including mitochondria [27,28,29,30,31]. As such, age-related mitochondrial dysfunction and Fe dyshomeostasis are advocated among the biological mechanisms involved in the pathogenesis of sarcopenia [12]. The existence of a mitochondrial–Fe feed-forward loop in the aging muscle has been hypothesized. This loop would involve perturbations in cellular Fe transport leading to Fe accrual, oxidative damage to mitochondrial DNA (mtDNA) and ETC complexes, together impinging on mitochondrial function and eventually generating a vicious circle that promotes further Fe overload and oxidative stress [12,32,33,34].

In light of the vital functions of mitochondria, the maintenance of organellar structural and functional integrity is essential for cell’s homeostasis and is ensured by an integrated network of pathways (i.e., mitochondrial dynamics, mitophagy, biogenesis, and proteostasis), collectively termed mitochondrial quality control (MQC) [35,36,37]. The MQC axis becomes impaired during aging, which is thought to trigger and amplify mitochondrial dysfunction [37,38]. As a result, pro-inflammatory routes may be triggered to dispose dysfunctional organelles [39,40,41,42,43,44]. 

In a recent work by our group, we provided an initial appraisal of the relationship between Fe imbalance, mitochondrial damage in muscle, and chronic inflammation in a cohort of older adults [32]. In that study, we observed that Fe import via TfR, a main Fe import protein, was markedly decreased in muscles of older people. Moreover, an association between Fe dyshomeostasis and systemic inflammation—i.e., higher circulating levels of hepcidin, interleukin 6, and C-reactive protein—emerged as a relevant factor possibly contributing to physical function impairment [32].

In subsequent work detailed in this manuscript, we further explored the relationship between Fe imbalance and mitochondrial dyshomeostasis in muscle biopsies obtained from the same cohort of individuals. Specifically, we sought to determine whether a differential distribution of Fe isotopes within the labile Fe pool and changes in the expression of proteins involved in mitochondrial Fe handling (i.e., mitoferrin and frataxin) and MQC in muscle would be associated with aging and declining physical performance.

## 2. Materials and Methods

### 2.1. Participants

Physically inactive community-dwelling men and women aged 70 years and older or between the ages of 18 and 35 years were included in the study. The Recruitment Core of the University of Florida Claude D. Pepper Older Americans Independence Center coordinated participants enrolment, as previously described [11,45,46]. A set of eligibility criteria, common for the two age groups, was chosen to minimize the possible confounding effect of comorbidities, medications, and lifestyle habits on the relationship among physical performance, Fe metabolism, and measures of muscle mitochondrial quality, as detailed elsewhere [32,47]. 

Older adults were categorized in HF and LF according to the summary score obtained on the Short Physical Performance Battery (SPPB) [48]. In particular, participants with an SPPB score ≥ 11 were classified as HF, while those who scored ≤ 7 were categorized as LF. The cut-off values were chosen for their ability to predict relevant health outcomes in older adults (e.g., functional limitations, institutionalization, mortality) [48,49,50,51,52]. To allow greater discrimination in physical function and biochemical parameters between the two groups, participants scoring 8–10 on the SPPB were not included.

The study protocol was approved by the University of Florida’s Institutional Review Board (IRB201300790) and all participants provided written informed consent prior to enrolment.

### 2.2. Collection of Muscle Biopsies

Muscle biopsies were obtained from the vastus lateralis of the dominant leg by percutaneous needle biopsy, under local anesthesia [46]. Upon collection, muscle specimens were cleaned of any visible blood and fat, snap-frozen in liquid nitrogen and stored at −80 °C until further processing and analysis.

### 2.3. Measurement of Iron Isotopes in Muscle Biopsies by Inductively Coupled Plasma-Mass Spectrometry 

All reagents used for the sample preparation and Fe ion-exchange purification were Optima-grade and the work was performed under Clean Lab (class 1000) environment. Between 0.013 g and 0.029 g of muscle tissue were digested for the determination of Fe concentration, following methods described elsewhere [32]. Briefly, tissues were digested in concentrated nitric acid (HNO_3_) and 30% hydrogen peroxide (H_2_O_2_) in pre-cleaned Teflon vials. A small fraction of the final solution was used for the determination of Fe concentration in muscle samples [32]. The remaining digested muscle solution was evaporated to dryness and converted to chlorides by 3× evaporation with 7 N hydrochloric acid (HCl). Fe was purified on an AG^®^ MP-1M resin (Bio-Rad Laboratories, Hercules, CA, USA) following methods described by Maréchal et al. [53]. Ion-exchange columns were packed with 2 mL of AG^®^ MP-1M (100–200 mesh) resin. After packing, the columns were washed with 0.5 N HNO_3_, followed by milliQ water, then 1 N HCl again followed by milliQ water. After the washing steps, the columns were equilibrated with 4 mL 7 N HCl. Samples were loaded in 7 N HCl with 0.001% H_2_O_2_. The matrix was eluted with 23 mL of 7 N HCl and Fe was collected in 10 mL 1 N HCl. The Fe fraction was evaporated to dryness. After evaporation, 0.5 mL of 8 N HNO_3_ were added to the dry residue and evaporated again. The samples were subsequently dissolved in 2% HNO_3_ for Fe isotope measurements. The Fe yield after column purification was 101% (±3%). Fe isotope measurements were performed on a Nu-Plasma 3 MC-ICP-MS (AMETEK, Wrexham, UK) in the high-resolution mode. Due to the low total Fe available, samples were introduced in the MC-ICP-MS via an Aridus3 desolvating nebulizer system (Teledyne CETAC, Omaha, NE, USA). All samples and standards were adjusted to 500 ppb Fe in the final solution, which resulted in around 6.5 V signal for ^56^Fe. Fe isotope measurements were performed on Faraday detectors in standard–sample–standard mode with IRM014 used as the bracketing standard. Fe isotope data are presented in delta-notation (δ) in parts per thousand (‰) using the following equation [20]:

δ^56^Fe = (^56^Fe/^54^Fe_(sample)_/^56^Fe/^54^Fe_(IRM014)_) × 1000 (1)

δ^57^Fe was calculated with Equation (1) using ^57^Fe/^54^Fe ratio instead of ^56^Fe/^54^Fe. Fe isotopes measured in BCR2 standard (*n* = 7), prepared and analyzed together with the samples, yielded the following results: δ^56^Fe = 0.097 (±0.014) and δ^57^Fe = 0.163 (±0.048). These values are within the error of published data for BCR2 by Craddock and Dauphas [54]: δ^56^Fe = 0.091 (±0.011) and δ^57^Fe = 0.126 (±0.017). 

### 2.4. Western Immunoblotting

Protein lysates were prepared from muscle biopsies and levels of beclin, microtubule-associated protein 1A/1B-light chain 3 (LC3B) I and II, frataxin, mitoferrin, sequestosome 1 (SQSTM1)/p62, Parkin, and phosphatase and tensin homolog-induced kinase 1 (Pink1) were measured by Western immunoblotting, as previously described [11]. A total of 50 µg of protein was separated on 4–20% polyacrylamide gels (Bio-Rad Laboratories), transferred onto polyvinylidene difluoride membranes (Bio-Rad Laboratories), and blocked for 1 h in 2.5% or 5% milk in Tris-buffered saline with Tween 20 (Bio-Rad Laboratories), as appropriate (Table 1). Bovine serum albumin- and milk-blocked blots were probed with commercially available primary and secondary antibodies listed in Table 1. Protein bands were detected with SuperSignal West Femto Maximum Sensitivity Substrate (Thermo Fisher Scientific, Waltham, MA, USA) using a ChemiDoc XRS imager (Bio-Rad Laboratories) and target bands were quantified using the Image Lab 6.0 software (Bio-Rad Laboratories). The “Total Lane Protein” setting was used for the quantification of target proteins. The spot density of each band was normalized to the total protein amount loaded in the same lane, as determined by the densitometric analysis of the corresponding Ponceau S-stained membranes [55,56].

### 2.5. Quantification of Mitochondrial DNA Deletion

Quantitative real-time polymerase chain reaction (qRT–PCR) was used to determine muscle levels of the mtDNA common deletion of 4977 bp (mtDNA^4977^) in total DNA purified as described elsewhere [57,58,59,60,61,62]. DNA purification was performed using a Wizard Genomic DNA Purification Kit (Promega, Madison, WI, USA) according to the manufacturer’s instructions. Briefly, 10–20 mg of muscle tissue were homogenized in 1 mL of nuclei cell lysis solution with a hard tissue disposable probe (Omni international, Kennesaw, GA, USA) on a PowerGen 500 homogenator (Thermo Fisher Scientific). DNA quantification was performed on a NanoDrop 1000 spectrophotometer (Thermo Fisher Scientific) and nucleic acid integrity was verified by gel electrophoresis on 0.8% agarose gel in 1× TBE (90 mM Tris-borate pH 7.4, 90 mM boric acid, 2.5 mM ethylenediaminetetraacetic acid). Forward and reverse primers for the assessment of mtDNA^4977^ were designed to span both ends of the deletion (forward 5′-CCTTACACTATTCCTCATCACC-3′; reverse 5′-TGTGGTCTTTGGAGTAGAAACC-3′; amplicon length: 127 bp) [62]. Primer pairs were checked for their specificity of binding to the mitochondrial genome by verifying the lack of cross-binding to nuclear-mtDNA sequences (numts) and by running melting curve analysis, non-template control reactions, and gel electrophoresis of PCR products. Glyceraldehyde 3-phosphate dehydrogenase (GAPDH) was used as an internal control (forward 5′-CAGAACATCATCCCTGCCTCTAC-3′; reverse 5′-TTGAAGTCAGAGGAGACCACCTG-3′; amplicon length 251 bp). Each sample was analyzed in triplicate in 20 µL final volume. The reaction mixture consisted of 1× Terra qPCR Direct SYBR Premix (Takara Bio USA Inc., Mountain View, CA, USA), 0.2 µM forward and reverse primers, and 10 ng of total DNA template. The amplification proceeded for 40 cycles. Amplification reactions were run on a CFX96 Touch™ Real-Time PCR Detection System (Bio-Rad Laboratories). The abundance of the mtDNA^4977^ deletion was calculated according to the Pfaffl mathematical model using the formula R = 2^ΔΔCt^ [63] and normalized to the relative mtDNA content. MtDNA relative quantification was obtained by RT–PCR using the Human Mitochondrial DNA Monitoring Primer Kit (Takara Bio USA). The primers amplified mitochondrial genes corresponding to the nicotinamide adenine dinucleotide reduced form (NADH) dehydrogenase subunits 1 and 5 (ND1, ND5) and nuclear genes corresponding to solute carrier organic anion transporter family, member 2b1 (SLCO2B1), and serpin family A member 1 (SERPINA1) [32]. Differences in threshold cycle values for the ND1/SLCO2B1 pair (ΔCt1 = Ct for SLCO2B1 − Ct for ND1) and the ND5/SERPINA1 pair (ΔCt2 = Ct for SERPINA1 − Ct for ND5) were calculated, and the average of 2^ΔCt^ for the values of ΔCt1 and ΔCt2 was used as a measure of relative mtDNA abundance.

### 2.6. Statistical Analysis

The normal distribution of data was verified via the Kolmogorov–Smirnov test. Normally distributed continuous variables were compared by one-way analysis of variance (ANOVA) followed by Tukey’s post-hoc test when appropriate. Differences for non-normally distributed continuous data were assessed by the non-parametric tests Mann–Whitney U and Kruskal–Wallis H with Dunns’ post-hoc test as appropriate. Differences in categorical variables among groups were determined via χ^2^ statistics. Relationships between variables were explored by linear regression analysis and the Pearson’s test. All analyses were performed using the GraphPrism 5.03 software (GraphPad Software, Inc., San Diego, CA, USA), with statistical significance set at *p* < 0.05.

## 3. Results

### 3.1. Study Participants

Thirty-four participants, 11 young (six men and five women; median age: 22 years; interquartile range: 21–28) and 23 older adults (14 men and nine women; median age: 80 years; interquartile range: 71–82) were included in the study. The main characteristics of study participants according to age and SPPB categories were previously reported [32] and are shown in Table 2. Sex distribution, body mass index, and the number of disease conditions and medications were comparable among groups. Age did not differ between the two subgroups of older adults. Consistent with the study protocol, HF participants had higher SPPB scores than LF older adults (*p* = 0.0002). Old participants showed a greater abundance of muscle total Fe levels independent of the SPPB score (*p* = 0.0256).

### 3.2. Measurement of Mitochondrial Iron Handling Proteins and Quantification of Iron Isotopes in Muscle Biopsies from Young and Old Participants

**Figure 1:**
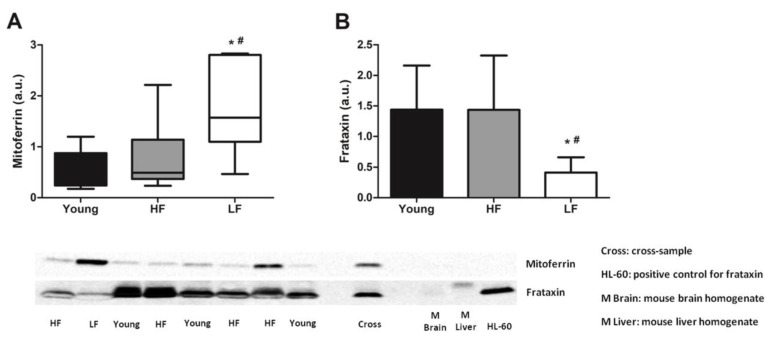
Protein levels of mitoferrin and frataxin in the vastus lateralis muscle of young and old participants. (**A**) Comparisons of mitoferrin levels by Kruskal–Wallis H statistics and of (**B**) frataxin by one-way ANOVA among young participants (*n* = 11) and high- (HF, *n* = 16) and low-functioning (LF, *n* = 7) older adults. In (**A**) box plots represent median values (interquartile ranges), while in (**B**) bars represent mean values (±standard deviation) for the three experimental groups. Values are expressed in arbitrary units (a.u.) and representative blots are shown for each protein. * *p* < 0.05 vs. young group; ^#^ *p* < 0.05 vs. HF group.

The expression of the two mitochondrial Fe handling proteins mitoferrin and frataxin were measured in muscle samples and evaluated for their association with age and physical performance categories. Mitoferrin levels were significantly higher in muscles of old LF participants compared with young and HF groups (*p* < 0.05; Figure 1A). Conversely, lower protein levels of frataxin were observed in LF older adults compared with young and HF participants (*p* < 0.05; Figure 1B).

**Figure 2:**
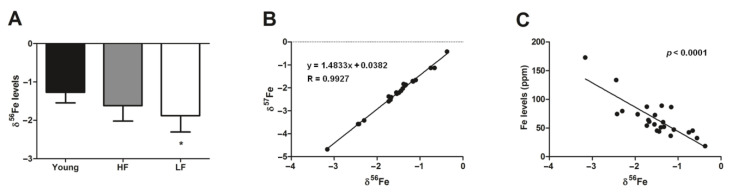
Iron isotope data for the vastus lateralis muscle of young and old participants. (**A**) Comparison by one-way ANOVA of δ^56^Fe among young participants (*n* = 11) and high- (HF, *n* = 16) and low-functioning (LF, *n* = 7) older adults. (**B**) Linear regression analysis between δ^57^Fe and δ^56^Fe in young and old participants. (**C**) Relationship between total Fe levels and δ^56^Fe in young and old participants as assessed by Pearson’s test. In (**A**) bars represent mean values (±standard deviation) for the three experimental groups. Values are expressed as ppm Fe. * *p* < 0.05 vs. young group.

To gain insights into the Fe status of muscles, we measured the Fe isotope composition across experimental groups. We then evaluated the relationship between δ^56^Fe and δ^57^Fe and between δ^56^Fe and total Fe levels in the whole study population. The ^56^Fe/^54^Fe and ^57^Fe/^54^Fe ratios follow a mass-dependent fractionation relationship (δ^56^Fe and δ^57^Fe; Figure 2B) indicating a mass-dependent Fe isotope fractionation. As expected, a value of 1.4848 was found for the slope of the linear trendline which is similar to the theoretical value of ln(57/54)/ln(56/54) ~ 1.487 [21]. The reduction of Fe^3+^ to Fe^2+^ leads to enrichment of the lighter isotope (^54^Fe) in the reduced fraction [20,21] and, according to Equation (1), lower abundance of δ^56^Fe and δ^57^Fe due to lower ^56^Fe/^54^Fe and ^57^Fe/^54^Fe relative to IRM014. As shown in Figure 2A, old LF participants showed lower abundance of δ^56^Fe compared with the young group (*p* = 0.0461). An inverse association was found between total Fe levels and δ^56^Fe abundance in the whole study sample (Figure 2C). The linear correlation detected between more negative δ^56^Fe and higher content of total Fe (Figure 2C) potentially indicates an increasing pool of labile Fe in cells with Fe overload.

### 3.3. Protein Levels of Selected Markers of Mitochondrial Quality Control and Abundance of the mtDNA^4977^ Common Deletion

**Figure 3:**
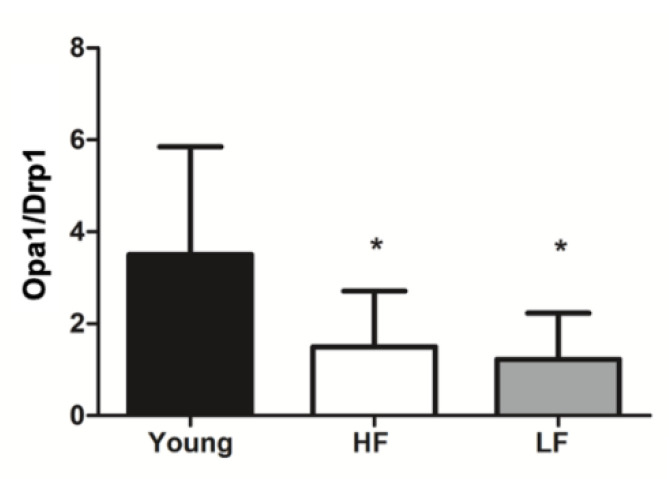
Mitochondrial fusion index in the vastus lateralis muscle of young and old participants. The fusion index was calculated as the ratio between protein levels of the fusion marker optic atrophy 1 (Opa1) and those of the fission factor dynamin-related protein 1 (Drp1). Differences among groups were assessed by one-way ANOVA. Bars represent mean values (±standard deviation) for the three experimental groups. * *p* < 0.05 vs. young group (*n* = 11); HF: high-functioning (*n* = 16); LF: low-functioning (*n* = 7).

As an indication of mitochondrial dynamics, we derived a fusion index from the ratio between protein levels of the fusion marker optic atrophy 1 and those of the fission factor dynamin-related protein 1 from previously published data in the same population [11]. The fusion index was lower in old versus young participants independent of SPPB scores (Figure 3).

**Figure 4:**
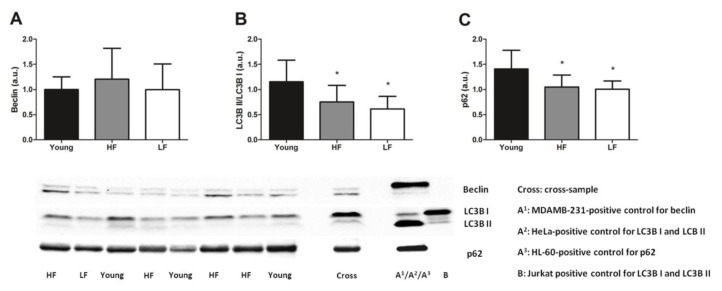
Selected markers of general autophagy in the vastus lateralis muscle of young and old participants. Comparison by one-way ANOVA of (**A**) protein levels of beclin, (**B**) ratio between lipidated (II) and non-lipidated (I) microtubule-associated protein 1A/1B-light chain 3 (LC3B), and (**C**) protein levels of p62 among young participants (*n* = 11) and high- (HF, *n* = 16) and low-functioning (LF, *n* = 7) older adults. Bars represent mean values (±standard deviation) for the three experimental groups. Values are expressed in arbitrary units (a.u.) and representative blots are shown for each protein. * *p* < 0.05 vs. young group.

Selected markers of general (i.e., beclin, LC3B, and p62) and mitochondrial autophagy (i.e., Pink1 and Parkin) were measured in muscle biopsies of all participants and related with age and physical performance. No differences among groups were found for the protein content of beclin (*p* = 0.5446, Figure 4A). However, the ratio between the lipidated (II) and the non-lipidated form (I) of LC3B and protein levels of p62, two markers of autophagic flux [64], were significantly lower in old participants independent of SPPB categories (*p* < 0.05, Figure 4B,C).

**Figure 5:**
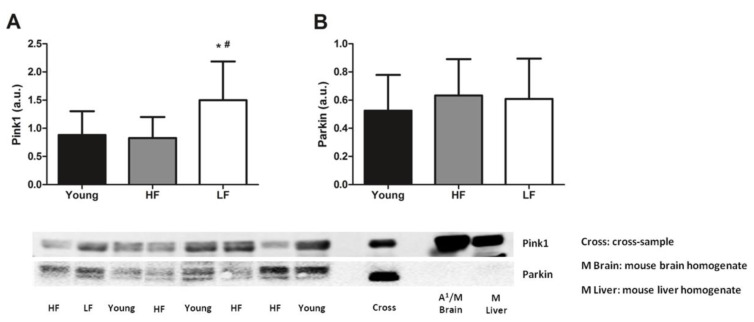
Protein levels of selected markers of mitophagy in the vastus lateralis muscle of young and old participants. Comparison by one-way ANOVA of (**A**) phosphatase and tensin homolog-induced kinase 1 (Pink1) and (**B**) Parkin among young participants (*n* = 11) and high- (HF, *n* = 16) and low-functioning (LF, *n* = 7) older adults. Bars represent mean values (± standard deviation) for the three experimental groups. Values are expressed in arbitrary units (a.u.) and representative blots are shown for each protein. * *p* < 0.05 vs. young group. ^#^ *p* < 0.05 vs. HF group.

As for the mitophagy markers assayed, higher protein levels of Pink1 were detected in old LF participants compared with young and HF participants (*p* < 0.05, Figure 5A). Protein levels of Parkin were instead unvaried among groups (*p* = 0.6338, Figure 5B).

**Figure 6:**
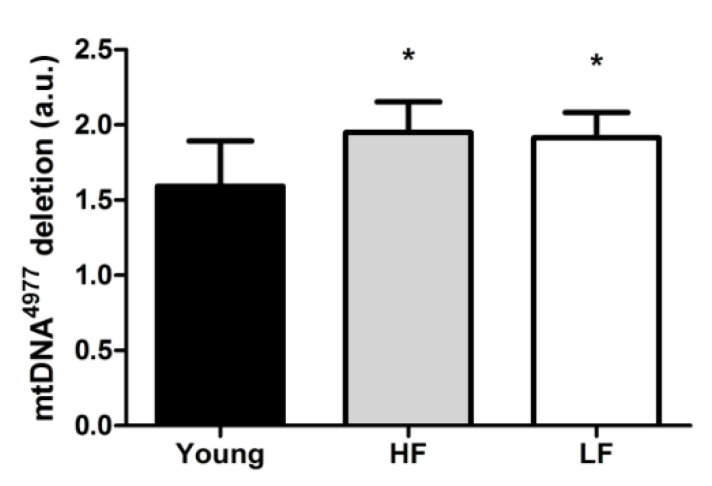
Relative abundance of the mtDNA^4977^ deletion in the vastus lateralis muscle of young and old participants. Differences among groups were assessed by one-way ANOVA. Bars represent mean values (±standard deviation) for the three experimental groups. Values are expressed in arbitrary units (a.u.). * *p* < 0.05 vs. young group (*n* = 11); HF: high-functioning (*n* = 16); LF: low-functioning (*n* = 7).

Finally, the relative abundance of the mtDNA common deletion (mtDNA^4977^) was quantified as a marker of mitochondrial damage. The abundance of such deletion was greater in old participants compared with the young group irrespective of the SPPB category (*p* < 0.05, Figure 6).

## 4. Discussion

The maintenance of skeletal muscle homeostasis relies upon the fine tuning of several processes, including ion balance [65,66,67]. Indeed, Fe dyshomeostasis is invoked as a factor contributing to age-associated muscle atrophy in rodents and humans through the mediation of oxidative stress [31,32]. A previous study by our group reported higher levels of non-heme Fe in muscles of old rats following hind limb suspension [31]. In rodent muscles, Fe overload was also shown to occur during aging and to be associated with RNA oxidative damage in subsarcolemmal mitochondria [33]. More recently, the relationship between muscular Fe status and age was also investigated in humans [32]. An age-dependent accumulation of non-heme Fe in muscle was found, which was associated with lower mtDNA content and greater mtDNA oxidative damage [32]. However, whether non-heme Fe accumulation in muscle with age is associated with excess labile Fe, as well as its relationship with changes in the expression of proteins involved in mitochondrial Fe handling and quality control, has remained unexplored. Hence, we sought to tackle this aspect of muscle Fe status by investigating whether the Fe isotope composition would be linked to MQC alterations during aging. We also explored whether labile Fe in muscle would be associated with physical performance.

In humans, the relative abundance of Fe isotopes (i.e., isotope fractionation) depends on redox conditions more than on ligand coordination [21], therefore reflecting the redox status of the surrounding milieu. In the scenario, an enrichment in Fe^2+^ is characterized by lower abundance of δ^56^Fe and δ^57^Fe [21]. Hence, old LF participants, in whom more negative levels of δ^56^Fe were observed, likely had higher levels of Fe^2+^ in muscle (Figure 2A). This finding, together with the observation of a higher expression of the mitochondrial Fe transporter mitoferrin and a lower content of the mitochondrial Fe storage protein frataxin in the same participant group (Figure 1A,B), suggests a possible dysregulation of mitochondrial Fe handling [68]. This idea is in keeping with the recent observation of increased intracellular labile Fe in conjunction with upregulation of mitoferrin in a mouse model of frataxin deficiency [68]. Mitoferrin upregulation in muscles of LF older adults might therefore be interpreted as a cell’s response to buffer Fe excess. This hypothesis is supported by the lower levels of frataxin detected in the same group, which might be indicative of inability of mitochondria to cope with Fe overload. This, in turn, would overwhelm the mitochondrial Fe storage capacity with most of the metal remaining unbound. The increased labile Fe content within mitochondria may compromise the organellar integrity via Fenton chemistry, as reflected by the greater abundance of mtDNA^4977^ deletion in old participants (Figure 6). 

**Figure 7:**
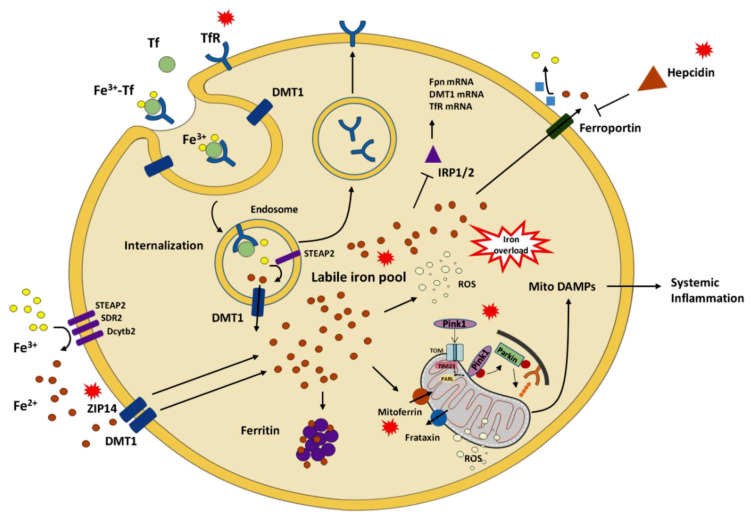
Schematic representation of putative pathways linking age-related systemic and muscular iron dyshomeostasis, derangements in mitochondrial quality control, and systemic inflammation. Red callouts indicate mediators/processes that are mainly dysregulated. Abbreviations: DAMPs, damage-associated molecular patterns; Dcytb2, duodenal cytochrome b2; DMT1, divalent metal transporter 1; Fpn, ferroportin; IRP1/2, iron-responsive element-binding protein 1/2; PARL, presenilin-associated rhomboid-like protein; Pink1, phosphatase and tensin homolog-induced kinase 1; ROS, reactive oxygen species; SDR2, stromal cell-derived receptor 2; STEAP2, six-transmembrane epithelial antigen of the prostate 2; Tf, transferrin; TfR, transferrin receptor; TIM23, translocase of inner mitochondrial membrane 23; TOM, translocase of the outer mitochondrial membrane; ZIP14, Zrt-Irt-like protein 14.

A shift of mitochondrial dynamics toward fusion is thought to serve as a mechanism to dilute mtDNA damage, including mtDNA^4977^ deletion, along the network, thereby avoiding its focal accumulation [38]. However, the fusion index, a surrogate indicator of the balance between mitochondrial fusion and fission, was lower in old compared with young participants (Figure 3). This finding could reflect the exhaustion of this compensatory mechanism in the aged muscle. Alternatively, a lower fusion index may indicate a shift toward fission as a response to extensive mitochondrial damage [38,69]. This idea is supported by the lower expression of frataxin in old LF participants which might represent an Fe-induced mitochondrial stress response to trigger Pink1/Parkin-dependent mitophagy for the disposal of damaged organelles [70]. However, this was not accompanied by an upregulation of the mitophagic flux, as suggested by decreased LC3B II/I (Figure 4B) and lower p62 levels (Figure 4C) in old participants. Collectively, these findings suggest that the clearance of damaged mitochondria via this route could not be achieved. Indeed, mitochondria primed for mitophagy by Pink1 might not be processed further as a consequence of possible clogging of this degradative pathway, as reflected by unvaried Parkin expression (Figure 5). Based on findings from the present study and those reported previously in the same participant cohort [32], an integrated set of molecular events linking age-related systemic and muscular Fe dyshomeostasis, MQC derangements, and systemic inflammation may be hypothesized as depicted in Figure 7.

The possibility that Fe mishandling in skeletal muscle might impact physical function through interfering with mitochondrial homeostasis and quality control opens new venues for interventions. Indeed, pharmacological compounds that are already in clinical use to treat a variety of Fe overload diseases (e.g., desferoxamine, deferasirox, deferiprone, salicylaldehyde isonicotinoyl hydrazone, alpha-lipoic acid) may be tested for their ability to scavenge and remove Fe from muscles. Such an approach is currently being explored to treat neurodegenerative diseases characterized by focal Fe accumulation, including Alzheimer’s disease, Parkinson’s disease, Friedreich’s ataxia, and amyotrophic lateral sclerosis [71]. An important issue related to Fe chelation therapy is that presently available compounds are not selective for organs or macromolecular structures. This may substantially limit their use in older adults, in whom iron-deficient anemia is highly prevalent [72]. Calorie restriction (CR) has been shown to mitigate age-associated Fe accumulation in various rodent tissues, including muscle [30]. However, the long-term implementation of this dietary regimen is hampered by feasibility and tolerability issues, especially in frail older adults [73]. This limitation may be overcome through the use of CR mimetics [73], among which resveratrol was found to attenuate the detrimental effects of Fe overload in preclinical models [74,75]. Whatever the strategy used, the perspective of preserving physical function in old age via ameliorating muscle Fe handling and mitochondrial homeostasis is worth exploration. In particular, studies are warranted to (1) definitely establish the role of Fe dysmetabolism in muscle aging and physical function decline, (2) determine the optimal timing for Fe scavenging therapies, and (3) identify noninvasively accessible markers of muscle Fe status to monitor the intervention.

## 5. Limitations

To our knowledge, this is the first study to investigate Fe isotope composition and the expression of mitochondrial Fe handling proteins, mitoferrin and frataxin, in human skeletal muscle. However, our work has some limitations that deserve discussion. First of all, the small sample size makes the study exploratory in nature and did not allow analyses to be adjusted for sex or other possible confounders. The limited amount of muscle tissue available narrowed our analyses to selected mediators of pathways pertaining to Fe handling and MQC mechanisms. Indeed, total and phosphorylated protein levels of other mitophagy-related factors, such as TANK-binding kinase 1, optineurin, nuclear dot protein 52, and ubiquitin could not be assessed, which prevented us from achieving a more comprehensive appraisal of the mitophagic process. The cross-sectional design of the study does not allow inferences to be made about the time course of changes of the analyzed factors and cause-effect relationships. For the same reason, no mechanistic hypotheses could be tested regarding the processes leading to Fe dyshomeostasis in muscle and its impact on mitochondrial health. Indeed, our data do not allow establishing whether Fe reduction and isotope fractionation occurred as a result of intracellular redox reactions or during Fe import within the cell. In the latter scenario, since the expression of TfR1 was previously found to be substantially downregulated in muscles of old LF participants [32], Fe isotope fractionation might have occurred during Fe import via divalent metal transporter 1 or Zrt-Irt-like protein 14. This point warrants further exploration through ad hoc designed investigations. Finally, the study did not include actively exercising participants. Although a physically inactive lifestyle was one of the eligibility criteria, this information was collected through self-report and no objective measure of physical activity was obtained. The lack of this information impeded the appreciation of possible effects of exercise or physical activity on Fe status, mtDNA^4977^ abundance, and the expression of MQC mediators in muscle. 

## Figures and Tables

**Table 1:** Technical specifications of the primary antibodies used for Western immunoblotting.

Antibody	Manufacturer and Catalog Number	Type	Species	Dilution	Blocking	Detected Band MW (kDa)
Beclin	Cell Signaling Technology (Beverly, MA, USA) (#3738S)	Polyclonal	Rabbit	1:2000	5% BSA TBS-t	60
Frataxin	Abcam (Cambridge, MA, USA) (ab110328)	Monoclonal	Mouse	1:500	5% milk TBS-t	23
LC3B	Cell Signaling Technology (#2775S)	Polyclonal	Rabbit	1:1000	5% BSA TBS-t	14 (LC3B II) 16 (LC3B I)
Mitoferrin	Abcam (ab102959)	Polyclonal	Rabbit	1:1000	5% milk TBS-t	37
SQSTM1/p62	Cell Signaling Technology (#5114S)	Polyclonal	Rabbit	1:2000	5% BSA TBS-t	62
Parkin	Abcam (ab77924)	Monoclonal	Mouse	2 μg/mL	2.5% milk TBS-t	52
Pink1	Abcam (ab23707)	Polyclonal	Rabbit	1:1000	5% milk TBS-t	66

Abbreviations: BSA, bovine serum abumin; LC3B, microtubule-associated protein 1A/1B-light chain 3; Pink1, phosphatase and tensin homolog-induced kinase 1; SQSTM1/p62, sequestosome 1/p62; TBS-t, Tris-buffered saline with Tween 20.

**Table 2:** Characteristics of study participants according to age groups and physical performance categories.

	Young (*n* = 11)	Old (*n* = 23)		
Characteristic		HF (*n* = 16)	LF (*n* = 7)	*p* Value
Age (years), median (IQR)	22 (21–28)	73 (71–81) *	80.0 (80–83) *	<0.0001 ^a^
Sex (women), *n* (%)	5 (45.5)	4 (25.0)	5 (71.4)	0.1076 ^b^
BMI (kg/m^2^), mean ± SD	24.9 ± 4.2	27.7 ± 3.6	27.8 ± 4.2	0.1604 ^c^
Total iron levels (ppm), median (IQR)	36.3 (32.9–58.8)	76.8 (54.0–111.3) *	82.9 (60.5–119.3) *	0.0256 ^c^
Number of diseases ^¥^, median (IQR)	1.0 (0–2.0)	1.5 (1.0–3.0)	2 (1.0–4.0)	0.1274 ^c^
Number of medications ^#^, median (IQR)	1.5 (0–3.0)	3.0 (1.0–8.0)	3.0 (0.5–5.5)	0.3112 ^c^
SPPB summary score, median (IQR)		11 (11–12)	6 (6–7)	0.0002 ^d^

Abbreviations: BMI, body mass index; HF, high-functioning; IQR, interquartile range; LF, low-functioning; SD, standard deviation; SPPB, short physical performance battery. * *p* < 0.05 vs. young group. ^¥^ includes hypertension, coronary artery disease, prior stroke, peripheral vascular disease, diabetes, chronic obstructive pulmonary disease, and osteoarthritis. ^#^ includes prescription and over-the-counter drugs. ^a^ Kruskal–Wallis H statistics. ^b^ χ^2^ test. ^c^ One-way ANOVA. ^d^ Mann–Whitney U test.
